# FSFT6mA: a feature-synthesis fine-tuning framework for DNA 6mA site prediction

**DOI:** 10.3389/fgene.2025.1750223

**Published:** 2026-01-12

**Authors:** Hong-Jin Yu, Ying Zhang, Dong-Jun Yu, Guansheng Zheng

**Affiliations:** 1 School of Computer Science, Nanjing University of Information Science and Technology, Nanjing, China; 2 School of Artificial Intelligence, Nanjing Normal University of Special Education, Nanjing, China; 3 School of Computer Science and Engineering, Nanjing University of Science and Technology, Nanjing, China

**Keywords:** deep learning, DNA 6mA prediction, generative adversarial network, sequence features, synthetic features

## Abstract

**Introduction:**

DNA N6-methyladenine (6mA) is an important epigenetic modification that plays a critical role in gene expression regulation and has been associated with diverse biological processes and diseases. Accurate identification of 6mA sites is essential for understanding its functional significance. Although an increasing number of computational approaches have been proposed, they almost exclusively rely on sequence-derived features. The potential of novel feature representations to further enhance predictive performance remains an important research problem.

**Methods:**

In this study, we propose FSFT6mA, a novel deep learning-based framework designed to improve 6mA site prediction through feature synthesis. The model is initially trained on the original datasets using a deep convolutional neural network. Subsequently, a Generative Adversarial Network (GAN) is employed to generate synthetic features from intermediate network layers, which are then used to fine-tune the well-trained model in the first stage.

**Results:**

Incorporating GAN-generated features leads to notable performance gains, improving *MCC* by 2.6% on *A. thaliana* and 1.9% on *D. melanogaster* compared with the base models without synthetic features. Independent validation experiments demonstrate that FSFT6mA achieves superior performance compared to existing state-of-the-art predictors, attaining *AUC* values of 0.969 and 0.968 on *A. thaliana* and *D. melanogaster*, respectively.

**Discussion:**

These results indicate that FSFT6mA is an accurate tool for DNA 6mA site prediction. The data and the codes used in this study are freely accessible on GitHub (https://github.com/YuHong-Jin/FSFT6mA).

## 1 Introduction

DNA modification is an important mechanism of epigenetic regulation, involving specific chemical alterations to nucleobases without changing the underlying genetic sequence (Liu Y. et al., 2021; Jin et al., 2011). Among various modifications, 5-methylcytosine (5mC) has been extensively investigated and is known for its essential roles in transcriptional regulation, embryonic development, and cellular differentiation (Ehrlich et al., 1981; Shi et al., 2017; Liu et al., 2019). In contrast, research on N6-methyladenine (6mA) has progressed relatively slowly. Initially, DNA 6mA was considered to occur exclusively in prokaryotes. However, with the development of advanced sequencing and detection technologies, 6mA has now been identified across all three domains of life: bacteria, archaea, and eukaryotes (Li et al., 2022; Wu, 2020; Liu X. et al., 2021; O’B et al., 2016). Interest in exploring the potential regulatory roles of 6mA in eukaryotic genomes has been increasing. Accumulating evidence indicates that 6mA is functionally significant in regulating gene expression, guiding development, and modulating diverse biological processes (Low et al., 2001; Fouse et al., 2010).

Although wet-lab techniques such as high-throughput immunoprecipitation sequencing (6mA-IP-seq) (Zheng et al., 2019; Fu et al., 2015; Liu et al., 2016) and single-molecule real-time (SMRT) sequencing (Zhang et al., 2023; Li et al., 2025) have substantially advanced the detection of 6mA sites, computational prediction approaches remain indispensable. These methods reduce the high experimental cost and associated demands, thereby complementing empirical studies and accelerating the understanding of the biological functions of 6mA.

A series of computational approaches for predicting DNA 6mA sites in eukaryotes have been proposed. iDNA6mA-PseKNC (Feng et al., 2019) is the first method for DNA 6mA site prediction, which is based on Support Vector Machine (SVM) and utilizes Pseudo K-tuple Nucleotide Composition (PseKNC) along with nucleotide physicochemical properties to achieve effective prediction. i6mA-Pred (Chen et al., 2019) utilizes SVM and incorporated nucleotide chemical properties along with nucleotide frequency as predictive features, reaching robust performance on the rice genome dataset. 6mA-RicePred (Huang et al., 2020) employs SVM along with a feature fusion method to combine advantageous features. Beyond SVM-based approaches, other traditional machine learning methods have also been applied. A bagging classifier is employed by iDNA6mA-Rice (Lv et al., 2019); Random Forest (RF) is employed by SDM6A (Basith et al., 2019) and 6mA-Finder (Xu et al., 2020); and a Markov model is employed by MM-6mAPred (Pian et al., 2020). These early computational approaches focus on the systematic design, selection, and integration of effective features, laying the groundwork for subsequent predictive models.

With the development of deep learning (Sharifani and Amini, 2023; Yousef and Allmer, 2023; Berrar and Dubitzky, 2021), many of these methods have been applied to the field of bioinformatics (Pham et al., 2024a; Pham et al., 2023), particularly for tasks such as RNA and DNA modification prediction (Pham et al., 2024b; Pham et al., 2024c). In the case of DNA 6mA site prediction, a variety of deep learning-based predictors have been proposed. For instance, DeepM6A (Tan et al., 2020) employs deep convolutional neural networks for DNA 6mA site prediction. SNNRice6mA (Yu and Dai, 2019) utilizes a lightweight deep learning model to identify DNA 6mA sites. Deep6mA (Li et al., 2021) integrates convolutional neural networks (CNN) with long short-term memory (LSTM) for prediction. LA6mA and AL6mA leverage self-attention mechanism and LSTM for 6mA site prediction. SNN6mA (Yu et al., 2023) uses a Siamese network to capture more discriminative features in a low-dimensional embedding space to improve performance. Notably, the methods described above depend exclusively on sequence-based features. Despite efforts to develop more informative sequence-based features, expanding the feature space for DNA 6mA prediction remains a topic of significant research interest.

Considering the limitation of existing computational methods in this field, we developed a new model, termed FSFT6mA (Feature-Synthesis Fine-Tuning for 6mA prediction). The overall procedure of FSFT6mA consists of two stages. In the first stage, a base model is trained on original sequence-derived features to learn the intrinsic representations of positive and negative samples. In the second stage, a generative adversarial network (GAN) is employed to synthesize additional features, which are then used to fine-tune the well-trained model from the first stage. This two-stage feature-synthesis fine-tuning strategy enhances feature diversity and improves the model’s generalization performance. Extensive experiments on benchmark datasets demonstrate that FSFT6mA achieves superior performance compared over existing approaches.

## 2 Materials and methods

### 2.1 Benchmark dataset

To evaluate the proposed FSFT6mA, we utilized DNA 6mA data from two benchmark datasets: *Arabidopsis thaliana* (*A. thaliana*) and *Drosophila melanogaster* (*D. melanogaster*) (Zhang et al., 2021). Table 1 summarizes the details of the datasets. The *A. thaliana* dataset contains 39,232 samples, while the *D. melanogaster* dataset includes 21,306 samples. Each sequence in both datasets is 41 bp in length. In the positive samples, a 6mA site is located in the middle of the sequence, whereas in the negative samples, the central position is a non-6mA site. Further details regarding dataset construction can be found in Zhang et al. (2021). The data were divided into training and independent test sets at a ratio of 9:1 by the reference study. We used the same partition to ensure a fair comparison. Additionally, in our training process, 1/9 of the training data was randomly chosen as the validation set.

**TABLE 1:** Details of benchmark datasets.

Species	Positive	Negative	Total
*A. thaliana*	19,616	19,616	39,232
*D. melanogaster*	10,653	10,653	21,306

### 2.2 Feature extraction

The dataset used in this study consists of DNA sequences, each of them composed of the four nucleotides ‘A’, ‘C’, ‘G’, and ‘T’. We employed a one-hot encoding scheme (Rodríguez et al., 2018) to convert each sequence into a numerical matrix. As shown in Equation 1, the following rules were adopted:

$$\begin{array}{l} A:1,0,0,0\\ \begin{array}{l} C:0,1,0,0\\ \begin{array}{l} G:0,0,1,0\\ T:0,0,0,1 \end{array} \end{array} \end{array}$$ (1)

According to the encoding rules, each sequence was transformed into a matrix of size 4×l , where l denotes the sequence length.

In addition to the basic one-hot encoding scheme, a feature-synthesis strategy was adopted to enrich the feature space, thereby enhancing the generalization ability of the original features. Specifically, features extracted from intermediate network layers of the model were further utilized to generate synthetic representations, which were separately learned from positive and negative samples. The aim of this process is to enhance the discriminative capacity of the model beyond the original feature distribution. The synthetic features were then reshaped to fit the network architectural requirements. This strategy leads to a two-stage learning framework, in which the model first learns base representations and is then further fine-tuned using synthesized features. Details of the feature-synthesis strategy are provided in the “Proposed Methodology” section.

### 2.3 Proposed methodology

**FIGURE 1:**
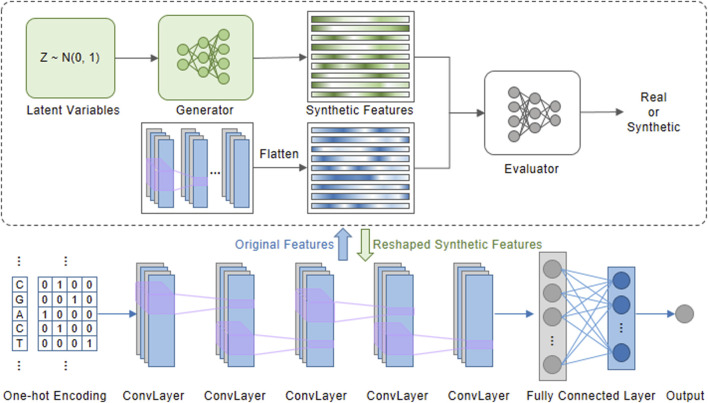
The overall framework of FSFT6mA designed for DNA 6mA prediction.

To overcome the limitations of models that directly rely on one-hot encoding, we propose FSFT6mA for predicting DNA 6mA sites, as illustrated in Figure 1. The overall procedure consists of two stages.

In the first stage, we constructed a deep convolutional neural network (Gu et al., 2018) to enable the model to distinguish between positive and negative samples, which was trained on the training set and validated on the validation set. FSFT6mA consists of five convolutional blocks followed by two fully connected layers and one sigmoid output layer. Each convolutional block consists of a convolutional layer, a LeakyReLU unit, and dropout regularization. We adopt an early stopping strategy in which the model is evaluated on the validation set after each training epoch, and a checkpoint is saved only when the validation AUC improves.

The convolutional layer can be expressed as Equation 2.

$$Conv{\left(X\right)}_{if}=\sum_{m=0}^{M-1}\sum_{n=0}^{N-1}W_{mn}^{f}X_{i+m,n}$$ (2)

where M and N denote the height and width of the convolutional kernel, respectively; $W^{f}$ is the convolution kernel weight of size M×N for the *f*-th kernel; X is the input of the convolutional layer.

The LeakyReLU and Sigmoid activation functions can be expressed as shown in Equations 3, 4.

$$LeakyReLU\left(x\right)=\left\{\begin{array}{l} x,\text{if }x\geq 0\\ ax,\text{if }x<0 \end{array}\right.$$ (3)

$$Sigmoid\;\left(x\right)=\frac{1}{1+e^{-x}}$$ (4)

where x denotes the input of a neuron, and a is a small positive constant.

In the second stage, features extracted from an intermediate convolutional layer were used to generate synthetic features by training a GAN. These features were then utilized to fine-tune the well-trained model from the first stage, enabling it to achieve improved performance and robustness in identifying 6mA sites.

According to reference (Wan and Jones, 2020), we adopted a Wasserstein Generative Adversarial Network with Gradient Penalty (WGAN-GP)-based strategy to generate synthetic features (Gulrajani et al., 2017). Specifically, the GAN framework consists of two core components: a generator and a discriminator. The generator learns to produce new features by mapping random noise and real features into a shared representation space. The discriminator distinguishes real features from generated features.

During the training process, the generator is optimized to produce synthetic samples that can effectively deceive the discriminator, while the discriminator is trained to accurately differentiate between real and generated samples (Goodfellow et al., 2020; Creswell et al., 2018). The objective function is defined as shown in Equation 5.

$$\min_{G}\underset{D}{\;\max}\;\underset{x∼P_{r}}{E}\left[\log\left(D\left(x\right)\right)\right]+\underset{z∼P_{g}}{E}\left[\log\left(1-D\left(G\left(z\right)\right)\right)\right]$$ (5)

where G and D denote the generator and discriminator, respectively, x represents real samples in data distribution $P_{r}$ , and z denotes random noise sampled from $P_{g}$ .

The output features extracted from an intermediate convolutional layer were flattened and used as real input data for the GAN. The synthetic features produced by the generator have the same dimensionality as the flattened inputs and the same number of samples as the validation set. During GAN training, synthetic features were generated at multiple training epochs. The similarity between real and synthetic features was quantitatively evaluated using a classifier two-sample test (CTST). Specifically, real features were labeled as 1 and synthetic features as 0, and then combined. The one-nearest-neighbour classifier with leave-one-out cross-validation was employed to distinguish between the two distributions. The CTST was computed every 200 GAN training epochs, and the epoch whose accuracy was closest to 0.5 was selected as the optimal synthesis features. After training, the produced synthetic feature vectors shared the distributional characteristics as the original features.

Once trained, these synthetic features can be further utilized for the second stage. The generated synthetic features were saved, reshaped to match the spatial dimensions of the corresponding convolutional layer, and subsequently used as additional inputs for model fine-tuning. During the fine-tuning stage, the first three convolutional layers were frozen to preserve the stability of the learned low-level representations, while only the parameters of the remaining layers were updated. Model optimization was performed using the Adam optimizer with a learning rate of 0.0001. The same early stopping strategy was adopted as that in the first stage.

## 3 Results and discussion

### 3.1 Evaluation metrics

To evaluate the performance of the proposed FSFT6mA, we used the training data to adjust the parameters and the testing data to evaluate the performance. Four evaluation metrics (Hossin and Sulaiman, 2015) were included, namely sensitivity (*Sen*), specificity (*Spe*), accuracy (*Acc*), and Matthews correlation coefficient (*MCC*), which are respectively defined in Equations 6–9.

$$Sen=\frac{TP}{TP+FN}$$ (6)

$$Spe=\frac{TN}{TN+FP}$$ (7)

$$Acc=\frac{TP+TN}{TP+FP+TN+FN}$$ (8)

$$MCC=\frac{TP\times TN-FP\times FN}{\sqrt{\left(TP+FP\right)\times \left(TN+FN\right)\times \left(TP+FN\right)\times \left(TN+FP\right)}}$$ (9)

where *TP*, *FP*, *TN*, and *FN* represent true positive, false positive, true negative and false negative, respectively. The values of *Sen*, *Spe*, *Acc* and range from 0 to 1, while *MCC* ranges between −1 and 1.

In addition, the Receiver Operating Characteristic (ROC) curve was employed for comprehensive evaluation, and the areas under the ROC curve (*AUC*) were calculated and provided.

### 3.2 Feature visualization and performance comparison

In this section, we evaluate FSFT6mA under two different conditions: the original model without fine-tuning (denoted as FSFT6mA-o) and the fine-tuned version of the model (denoted as FSFT6mA).

Firstly, we compared the performance of FSFT6mA-o and FSFT6mA on the test datasets, as summarized in Table 2. FSFT6mA achieved higher values of *Spe*, *Acc*, *MCC*, and *AUC* than FSFT6mA-o. Specifically, FSFT6mA-o achieved *AUC* values of 0.962 and 0.966 on *A. thaliana* and *D. melanogaster*, respectively, while FSFT6mA improved the *AUC* values to 0.969 and 0.968, respectively. Furthermore, at a fixed threshold, FSFT6mA exhibited a notable improvement over FSFT6mA-o in terms of *MCC*. These results demonstrate the effectiveness of the fine-tuning process.

**TABLE 2:** Performance comparison between FSFT6mA-o and FSFT6mA on the test datasets.

Datasets	Method	*Sen*	*Spe*	*Acc*	*MCC*	*AUC*
*A. thaliana*	FSFT6mA-o	0.900	0.910	0.905	0.811	0.962
	FSFT6mA	0.899	0.933	0.916	0.832	0.969
*D. melanogaster*	FSFT6mA-o	0.904	0.920	0.912	0.825	0.966
	FSFT6mA	0.898	0.941	0.920	0.841	0.968

FSFT6mA-o, original model without fine-tuning; FSFT6mA, fine-tuned version of the original model.

**FIGURE 2:**
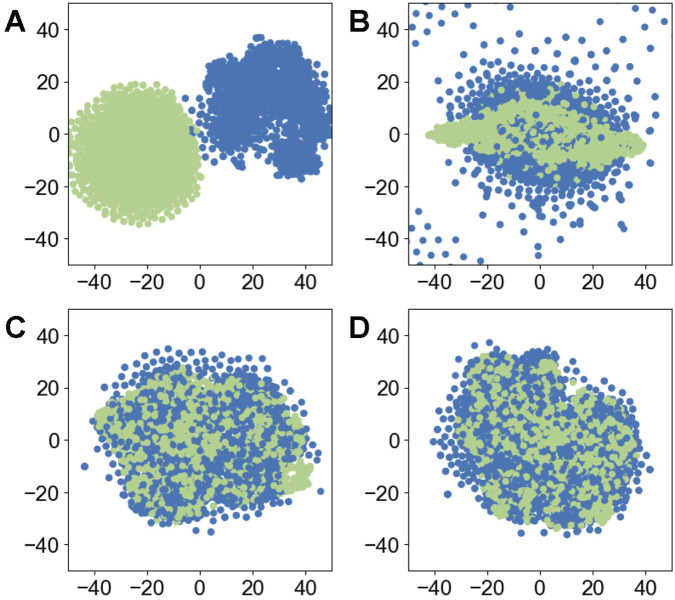
t-SNE visualization of real features at different training epochs: **(A)** epoch 0, **(B)** epoch 600, **(C)** epoch 1,200, and **(D)** epoch 1,800. Blue and green points represent real positive and synthetic positive features, respectively.

**FIGURE 3:**
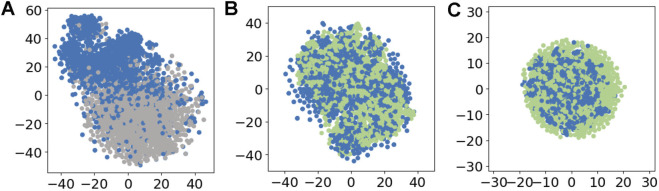
t-SNE visualization of features: **(A)** real positive (blue) vs. real negative (gray) features, **(B)** real positive (blue) vs. synthetic positive (green) features, and **(C)** real negative (blue) vs. synthetic negative (green) features.

To explore the feature representations learned by FSFT6mA-o and the synthetic features generated by GAN, we employed t-distributed stochastic neighbor embedding (t-SNE) for visualization, as shown in Figures 2, 3. Figure 2 illustrates that, as the number of GAN training epochs increases, the distribution of synthetic samples becomes increasingly similar to that of the real samples. Figure 3A illustrates that FSFT6mA-o effectively separates features derived from positive and negative samples, demonstrating its capability to discriminate between samples with different labels. As shown in Figures 3B,C, the synthetic features generated by the GAN are highly similar to real ones across both positive and negative samples. During fine-tuning, this strategy allows the model to integrate the synthetic features more efficiently, thereby enhancing the robustness of feature representations and improving overall performance.

In summary, these results confirm that GAN is effective and that fine-tuning enhances the model’s overall prediction performance.

### 3.3 Comparison with existing predictors

To evaluate the effectiveness of the proposed FSFT6mA, we compared it with five DNA 6mA prediction methods, including LA6mA (Zhang et al., 2021), AL6mA (Zhang et al., 2021), i6mA-DNC (Park et al., 2020), iDNA6mA (Tahir et al., 2019), and 3-mer-LR (Zhang et al., 2021) on the benchmark datasets. Among them, the first four are deep learning-based methods, whereas the last one is based on logistic regression (LR). All models were trained on the same training datasets and evaluated on the same test datasets to ensure a fair comparison.

**FIGURE 4:**
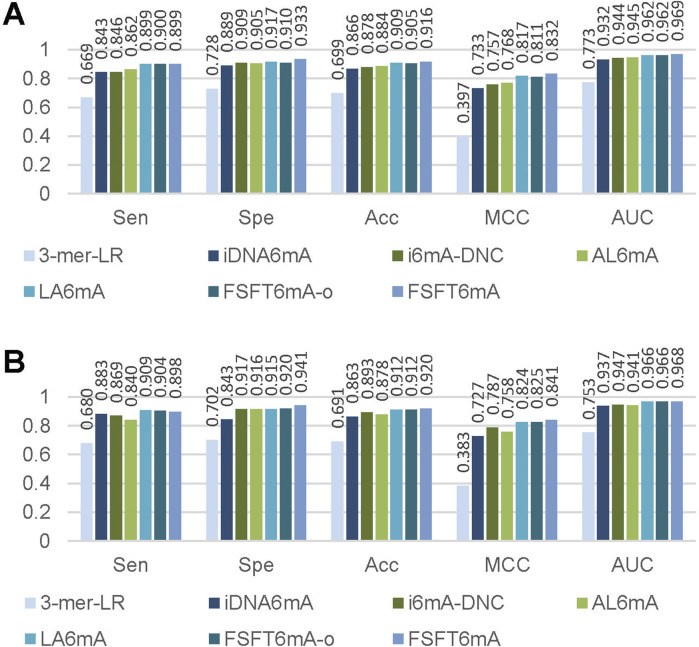
Performance comparison of FSFT6mA, FSFT6mA-o and five compared methods on the test datasets of **(A)** *A. thaliana* and **(B)** *D. melanogaster*.

**FIGURE 5:**
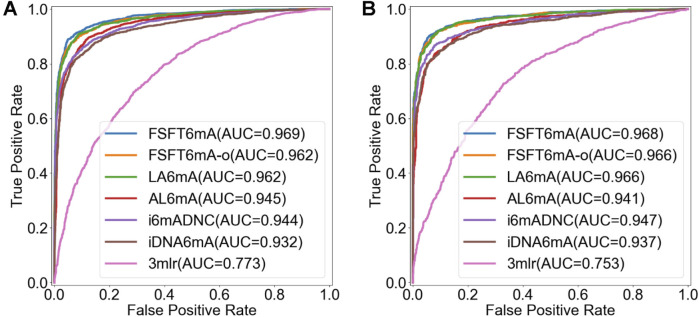
The ROC curves on the test datasets of **(A)** *A. thaliana* and **(B)** *D. melanogaster*.

The results and performance were summarized in Figures 4, 5. Figure 4 presents the bar charts of prediction performance on the two datasets. Among these methods, 3-mer-LR performs significantly worse than the deep learning-based models on both datasets with *AUC* values of 0.773 and 0.753 on *A. thaliana* and *D. melanogaster*, respectively. Deep learning-based models generally outperform traditional machine learning methods. The proposed FSFT6mA achieves the best overall performance with *AUC* values of 0.969 and 0.968 on *A. thaliana* and *D. melanogaster*, respectively. Figure 5 illustrates the ROC curve comparison of different methods. It can be seen that the proposed FSFT6mA provides better discrimination capability than the compared methods.

## 4 Conclusion

Accurate identification of DNA 6mA sites is of great importance for downstream analyses in the field of bioinformatics. In this study, we proposed FSFT6mA, a novel feature-synthesis fine-tuning framework for DNA 6mA site prediction. FSFT6mA used the training data to train a model firstly. Then, a GAN is employed to generate synthetic features, which were used to fine-tune the model and had been proven to be capable of improving performance.

The proposed approach demonstrates promising generalizability to other classification tasks in bioinformatics and computational biology. Moreover, it provides a potential strategy for few-shot learning tasks.

## Data Availability

Publicly available datasets were analyzed in this study. This data can be found here: https://github.com/YuHong-Jin/FSFT6mA.
